# Creation of an innovative diagnostic framework for hepatocellular carcinoma employing bioinformatics techniques focused on senescence-related and pyroptosis-related genes

**DOI:** 10.3389/fonc.2025.1485421

**Published:** 2025-02-13

**Authors:** Baixue Liu, Youguang Ao, Chunhui Liu, Feiyun Bai, Zhi Zhou, Juan Huang, Qi Wang

**Affiliations:** ^1^ College of Traditional Chinese Medicine, Inner Mongolia Medical University, Hohhot, China; ^2^ Hepatology Department, Ordos Second People’s Hospital, Ordos, China; ^3^ Department of Traditional Chinese Medicine, Inner Mongolia Autonomous Region Hospital of Traditional Chinese Medicine, Hohhot, China

**Keywords:** hepatocellular carcinoma, bioinformatics techniques, senescence, pyroptosis, diagnostic framework

## Abstract

**Background:**

Liver hepatocellular carcinoma (LIHC) continues to pose a major global health concern and is characterized by elevated mortality rates and a lack of effective therapies. This study aimed to explore differential gene expression linked to cellular senescence and pyroptosis in LIHC and to develop a prognostic risk model for use in clinical settings.

**Methods:**

We acquired datasets from The Cancer Genome Atlas (TCGA) and Gene Expression Omnibus (GEO). DESeq2 was used to identify differentially expressed genes associated with cell senescence and pyrodeath. The least absolute shrinkage and selection operator (LASSO) regression model was developed using cellular senescence- and pyroptosis-related differentially expressed genes (CSR&PRDEGs), and its predictive performance was evaluated with Kaplan–Meier survival analysis and time-dependent receiver operating characteristic (ROC) curves. We also performed various functional analyses of the genes. These findings were validated by real-time fluorescence quantitative polymerase chain reaction (PCR).

**Results:**

Using bioinformatics analysis, we developed a prognostic risk framework incorporating six critical genes: *ANXA2, APOA1, EZH2, IGF2BP3, SQSTM1*, and *TNFRSF11B*.The model demonstrated a statistically significant difference in overall survival between the high-risk and low-risk groups (p < 0.05). Additionally, real-time fluorescence quantitative PCR confirmed that genes *ANXA2, APOA1, EZH2, IGF2BP3, SQSTM1*, and *TNFRSF11B* were significantly overexpressed in the peripheral blood of patients with LIHC in comparison to normal volunteers, thereby validating the prognostic risk model’s accuracy.

**Conclusions:**

This study systematically elucidated the functions of genes associated with senescence and pyroptosis in LIHC cells. The constructed prognostic risk model serves to guide the development of personalized treatment plans, enhance patient management via risk stratification, facilitate the identification of high-risk patients, intensify monitoring or implement proactive interventions, thereby providing a novel perspective for the diagnosis and treatment of LIHC.

## 1 Introduction

Hepatocellular carcinoma (LIHC) is the fourth most common cause of cancer-related deaths worldwide, with approximately 782,000 new cases and 746,000 fatalities annually (1). The onset of LIHC is insidious, its etiology is multifaceted, early detection is challenging, and treatment options are limited. The current treatment approaches include surgery, liver transplantation, immunotherapy, targeted therapy, radiotherapy, and chemotherapy (2). Surgical intervention remains the preferred method, offering superior prognostic outcomes compared with other treatments, with a significant increase in the 5-year survival rate after surgical resection, reaching nearly 80% over the past decade (3). However, this approach requires early diagnosis and is ineffective against subclinical metastasis (4). Liver transplantation is the most effective treatment for end-stage LIHC. However, its broad application is limited by the shortage of donor organs (5). Although immunotherapy and targeted therapy are advantageous because of their convenience and precise therapeutic effects, they are accompanied by high toxicity and side effects (6, 7). Transcatheter arterial chemoembolization can effectively target tumor cells; however, it also leads to gastrointestinal side effects due to the chemotherapy drugs, bone marrow suppression, and post-embolization syndrome (8). Radiofrequency ablation is limited to small, non-metastatic tumors and may result in tumor recurrence or residual tumor after resection as well as potential liver function impairment (9, 10). For individuals with advanced LIHC, the 5-year survival rate is less than 15%. This highlights the pressing need for novel diagnostic and prognostic biomarkers for early detection and diagnosis (11).

According to the emerging “cancer evolution and development” theory (12), cancer arises from the interplay between innate genetic factors and acquired environmental influences such as viral infections, which disrupt immune balance or function, leading to persistent, uncontrollable inflammation. Within this inflammatory immune microenvironment, a small subset of “driver variant” cells that control cell proliferation, differentiation, and apoptosis, and promote tumorigenesis, are selected and expand. These cells gradually evolve into tumor-initiating cells with stem cell-like properties. This theory underscores that in an inflammatory microenvironment, cells undergo an evolutionary process of “variation - selection - adaptation,” ultimately leading to cancer. Cell senescence and pyroptosis are two crucial biological processes involved in tumor inflammation. Cell senescence, characterized by stable cell cycle arrest, can inhibit tumor growth by halting the proliferation of damaged cells (13). However, senescent cells may also create a pro-inflammatory microenvironment that fosters tumorigenesis (14). Pyroptosis, a form of programmed cell death characterized by an inflammatory reaction, can either inhibit or promote tumor growth depending on the surrounding environment (15). An intricate balance between these processes and their influence on cancer development and progression has been noted in various cancers (16); however, their role in LIHC remains underexplored.

Currently, diagnosing LIHC using alpha-fetoprotein combined with abdominal ultrasound screening does not allow for early analysis and diagnosis, and does not fully meet clinical diagnostic needs. To address this, our study employed a variety of bioinformatics analysis techniques, integrating multiple datasets, to comprehensively investigate the relationship between senescent cells and pyroptosis in LIHC. The aim was to develop a predictive risk model based on cellular senescence- and pyroptosis-related differentially expressed genes (CS&PRDEGs) and to determine novel tumor markers to enhance and supplement existing screening strategies. This approach aimed to improve the prognostic capability of patients with liver cancer and explore the biological functions and potential mechanisms of these genes in LIHC.

## 2 Materials and methods

### 2.1 Data

LIHC data were acquired from The Cancer Genome Atlas (TCGA) using the R package TCGAbiolinks (Version 2.30.0) (17). After removing samples without clinical information, sequencing data in count format for 373 LIHC samples, along with their survival outcomes and times, and 50 normal samples were collected. Data were standardized to fragments per kilobase per million (FPKM). Data for clinical studies were collected from the UCSC Xena database (18) (https://xena.ucsc.edu/). For more detailed information, please refer to **Table 1**.

**Table 1:** Baseline table with TCGA-LIHC patients characteristics.

Characteristics	Overall
n	373
Age (%)	
<= 60	177 (47.5)
> 60	196 (52.5)
Gender (%)	
FEMALE	121 (32.4)
MALE	252 (67.6)
Stage (%)	
Stage I	173 (49.6)
Stage II	86 (24.6)
Stage III	85 (24.4)
Stage IV	5 (1.4)

TCGA, The Cancer Genome Atlas; LIHC, Liver Hepatocellular Carcinoma.

The LIHC datasets GSE84402 (19) and GSE46408 (20) were retrieved from the Gene Expression Omnibus (GEO) database (https://www.ncbi.nlm.nih.gov/geo/) using the R package GEOquery (Version 2.70.0) (21). Both datasets originated from human liver tissue, these datasets encompass gene expression profiles for both LIHC and normal samples. Upon downloading, the data undergo an initial quality control check to ensure the absence of missing values and proper formatting for subsequent analysis. The microarray platforms used were GPL570 for GSE84402 and GPL4133 for GSE46408. Detailed information is presented in **Table 2**. The GSE84402 dataset included 14 LIHC and 14 normal samples, whereas the GSE46408 dataset included 6 LIHC and 6 normal samples. This study included all the LIHC and normal samples from these datasets.

**Table 2:** GEO Microarray Chip Information.

	GSE84402	GSE46408
Platform	GPL570	GPL4133
Type	Array	Array
Species	Homo sapiens	Homo sapiens
Tissue	Liver	Liver
Samples in LIHC group	14	6
Samples in Normal group	14	6
Reference	PMID: 28810927	PMID: 23922981

GEO, Gene Expression Omnibus.

We collected cellular senescence-related genes (CSRGs) and cell aging-related genes from the GeneCards database (22) (https://www.genecards.org/) and relevant published literature. Employing “cellular senescence” as the search keyword, we filtered for genes related to cellular senescence with “protein coding” status and a “relevance score > 0.” This search yielded 3575 CSRGs. Similarly, using the same keyword in the PubMed database (https://pubmed.ncbi.nlm.nih.gov/), we identified 279 cell senescence genes in the published literature (23). After merging datasets and eliminating duplicates, we identified a total of 3609 CSRGs. **Supplementary Table S1** provides detailed information.

Pyroptosis-related genes (PRGs) were obtained from the GeneCards database and published literature. By employing the term “pyroptosis” and applying a filter for “protein coding” genes that had a “relevance score > 0,” we identified 502 genes associated with pyroptosis. Furthermore, a keyword search for “pyroptosis” in PubMed yielded 33 genes (24). After consolidating and removing duplicates, we identified 510 PRGs. Detailed information is provided in **Supplementary Table S2**. The intersection of CSRGs and PRGs revealed 311 genes common to both categories; further details are available in **Supplementary Table S3**.

To correct for batch effects, the GSE84402 and GSE46408 datasets were processed utilizing the ComBat function from the sva package in R (Version 3.5.0) (25). The function corrects for both known and unknown batch effects within the datasets, enhancing data integration from diverse experimental conditions or time points, resulting in a combined GEO dataset of 20 LIHC samples and 20 normal samples. While the SVA approach significantly reduces batch effects, some systematic biases may remain. To further minimize these influences and ensure accuracy and reliability, we will implement multiple validation strategies and optimize data integration methods. The R package limma (Version 3.58.1) (26) standardizes the integrated GEO dataset, mapping gene probes to their corresponding genes based on the latest annotation for biological relevance. Normalization is performed to ensure comparability of expression intensities across samples, reducing technical variability. Principal component analysis (PCA) (27) is conducted on expression matrices before and after batch effect correction to evaluate the adjustments’ effectiveness, allowing visualization of clustering and differences between samples in 2D or 3D plots.

### 2.2 Differentially expressed genes associated with cellular senescence and pyroptosis in hepatocellular carcinoma

In the hepatocellular carcinoma dataset (TCGA-LIHC), samples were categorized into two groups: LIHC and normal. The R package DESeq2 (Version 1.42.0)was used to compare the two groups. Genes exhibiting differential expression (DEGs) were identified using |logFC| > 1 and adjusted to p < 0.05. Genes with logFC > 1 and adjusted p < 0.05 were recognized to have increased expression, whereas those with logFC < -1 and adjusted p < 0.05 were classified as downregulated. The p-value was adjusted using the Benjamini-Hochberg method. The results of the variable expression analysis are depicted using a volcano plot constructed using the R package ggplot2 (Version: 3.4.4).

To identify cellular senescence- and pyroptosis-related differentially expressed genes (CSR&PRDEGs) in hepatocellular carcinoma, variance analysis was performed on all DEGs that complied with the standards of |logFC| > 1 and were adjusted to p < 0.05. By intersecting these with CSRGs and PRGs, we mapped a Venn diagram to identify genes with altered expression associated with both cellular senescence and cell pyroptosis (CSR&PRDEGs).

### 2.3 Creation of a prognostic risk model for hepatocellular carcinoma

To create a risk prediction model for the TCGA-LIHC dataset, we employed the R package glmnet (Version 4.1-8) (28) to performed least absolute shrinkage and selection operator (LASSO) regression analysis. This examination was used for the CSR&PRDEGs identified through univariate Cox regression, utilizing the “Cox” family parameter and conducting 5-fold cross-validation with 10 iterations. LASSO regression improves linear regression by incorporating a penalty term (lambda × absolute value of the coefficient), which helps decrease model overfitting and increases the generalizability of the model. In this study, we optimized the hyperparameter lambda of the LASSO regression model using a 5-fold cross-validation technique to ensure robustness. Each fold involved evaluating multiple candidate lambda values and recording performance metrics, allowing us to select the optimal lambda that maximizes model performance. We further employed tenfold repeated cross-validation to enhance the reliability of the validation outcomes. Additionally, we generated LASSO model plots and variable trajectories to visualize the model selection process, providing clearer insights into model and feature dependencies. The outcomes of the LASSO regression were depicted in a prognostic risk model diagram and variable trajectory plot, facilitating the identification of genes relevant to the risk prediction framework. Subsequently, the LASSO risk score was calculated as follows:


$$riskScore=\sum_{i}Coefficient\;\left(gene_{i}\right)\;*\;mRNAExpression\;\left(gene_{i}\right)$$

Next, samples of LIHC from TCGA-LIHC dataset were grouped into high- and low-risk groups based on the median LASSO risk score obtained from the risk prediction model.

### 2.4 Prognostic analysis and validation of hepatocellular carcinoma prognostic risk model

To assess variations in overall survival (OS) between the high- and low-risk groups of LIHC samples from TCGA-LIHC dataset, Kaplan–Meier (KM) curve analysis was performed using the R package survival (Version 3.5-7) (29). The Kaplan–Meier curves were constructed based on the LASSO risk score. We processed all included samples with censored data to ensure accurate recording of the survival time and status for all patients. Patients who did not reach the event endpoint during follow-up were categorized as having censored data. We refrained from interpolating survival times to preserve the integrity and authenticity of the data. To ensure the accuracy and reliability of our analysis, we utilized survival data associated with the LASSO risk score. During the analysis, we screened all samples with complete clinical information, including only those patients with documented survival outcomes and survival times. This approach minimizes potential bias and ensures the validity of our Kaplan-Meier analysis. Furthermore, time-dependent ROC curve analysis (30) was employed to assess the model performance, identify the optimal model, and determine the best threshold. The timeROC R package (Version 0.4) was used to generate time-dependent ROC curves and compute the area under the ROC curve (AUC). This analysis utilized the LASSO risk score and OS data to predict 1-year survival rates for the LIHC samples, as well as survival outcomes for 3- and 5-years. The AUC value varies between 0.5 and 1, where a higher AUC suggests better diagnostic performance. An AUC above 0.5 signifies some predictive ability; values ranging from 0.5 to 0.7 suggest low precision, those between 0.7 and 0.9 signify moderate accuracy, and values exceeding 0.9 denote high accuracy.

To investigate the relationship between the risk Score and prognosis, and to assess its prognostic value, univariate and multivariate Cox regression examinations were conducted using variables such as risk score, sex, age, and clinical stage from the LASSO model. The findings of these analyses were depicted using a forest plot. Furthermore, we developed a nomogram (31), which is a graphical tool that represents the influence of multiple independent factors, using a set of disconnected line segments on a rectangular coordinate system. Employing the R package rms (Version 6.7-1), we constructed a nomogram based on multivariate Cox regression results to illustrate the connection between the risk score and clinical variables, offering predictions for survival outcomes at 1-, 3-, and 5-years.

To evaluate the model’s prediction precision, we employed a calibration curve to compare the predicted probabilities with the actual outcomes under various conditions. Calibration was performed to assess the precision and reliability of the prognostic risk model that integrated the LASSO risk score and clinical data. Decision curve analysis (DCA) is a simple technique used to evaluate clinical prediction models, diagnostic tests, and molecular markers. We used the R package ggDCA (Version 1.1) to generate a DCA plot based on the nomogram, evaluating the precision and discriminative power of the prognostic risk model for forecasting survival outcomes at 1-, 3-, and 5-years for LIHC.

### 2.5 Gene ontology and pathway enrichment analysis

Gene Ontology (GO) analysis (https://www.geneontology.org/) (32) is widely utilized for functional enrichment studies and encompasses categories such as biological processes, cellular components, and molecular functions. The Kyoto Encyclopedia of Genes and Genomes (KEGG) (https://www.genome.jp/kegg/) (33) provides a comprehensive database of genomic data, biological pathways, diseases, and drugs. In this study, the R package clusterProfiler (34) was used to conduct GO and KEGG pathway enrichment analyses of the genes within the risk prediction model. Statistical significance was determined using an adjusted p-value threshold of less than 0.05 and a false discovery rate (FDR) threshold of less than 0.25. The Benjamini-Hochberg procedure was employed for p-value adjustment.

### 2.6 Gene set enrichment analysis for high- and low-risk groups

In the TCGA-LIHC dataset, samples were classified into high- and low-risk groups based on the median LASSO risk scores. Gene set enrichment analysis (GSEA) was performed utilizing the R package `clusterProfiler`(Version 4.10.0) on all genes within these LIHC samples. For GSEA, the analysis parameters included setting a random seed of 2023, with a range of ten five 500 genes within each gene set. The Molecular Signatures Database (MSigDB) was accessed for C2 gene sets, specifically the cp.all.V2022.1.Hs.symbols.gmt dataset, encompassing all canonical pathways (3050 sets). GSEA findings were deemed significant if the adjusted p-value was < 0.05, and the false discovery rate was < 0.25, with p-value adjustment performed using the Benjamini-Hochberg method.

### 2.7 Gene set variation analysis for high- and low-risk groups

Gene set variation analysis (GSVA) (35) is a nonparametric, unsupervised technique that evaluates gene set enrichment by transforming individual gene expression matrices across samples into matrices representing gene sets. This method determines whether specific pathways are significantly enriched in various samples. In this study, gene sets were obtained from the MSigDB database, specifically the h.all.v2023.2.hs.symbols.gmt file. GSVA was performed on the TCGA-LIHC dataset to investigate gene mutations and variations in functional enrichment analysis, contrasting groups with high and low risks. Significant findings were identified with an adjusted p-value of < 0.05, and p-values were adjusted using the Benjamini-Hochberg method.

### 2.8 Protein interaction network and gene expression differences

The protein-protein interaction (PPI) network comprises proteins that engage with each other, affecting different biological activities, such as gene regulation, metabolism, signaling, and cell cycle control. Studying these interactions is essential for comprehending how proteins operate within biological systems, contribute to signaling pathways, and influence cellular functions and metabolism, particularly under disease conditions. The STRING database (36) (https://string-db.org/) provides information on recognized and predicted interactions between proteins. In this study, we used the STRING database to analyze interactions involving genes from a prognostic risk model. To build the PPI network related to these genes, We selected a minimum interaction coefficient threshold of greater than 0.150, which corresponds to a low confidence level. This network aids in the identification of molecular complexes with specific biological functions based on tightly connected regions. Genes exhibiting significant interactions in the PPI network were selected for further analysis.

To evaluate the variations in gene expression of prognostic risk model genes between the LIHC and normal groups, we used the Mann–Whitney U test. We generated comparison charts to depict the expression levels of these genes within the TCGA-LIHC dataset and across the combined datasets.

### 2.9 Quantitative real-time reverse transcription polymerase chain reaction analysis

Peripheral blood samples were obtained from six patients with LIHC and six healthy controls from the Inner Mongolia Hospital of Traditional Chinese Medicine. The inclusion criteria required that patients meet the latest guidelines of the US National Comprehensive Cancer Network for Hepatobiliary Tumors, Version 2.2021 (specifically for liver cancer), excluding patients with metastatic liver cancer. Healthy volunteers without cognitive impairments were included in the control group. Informed consent was obtained from all participants, and the study was approved by the Medical Ethics Committee of Inner Mongolia Medical University (Approval No.: YKD202402165). RNA was extracted from peripheral blood samples using the TRIzol reagent (G3013, SERVICEBIO, CHINA). RNA quality and integrity were thoroughly assessed before qPCR experiments. Purity was evaluated by measuring the absorbance at 260 nm and 280 nm using a spectrophotometer, resulting in an A260/A280 ratio of 1.8 to 2.0, indicating high purity, and an A260/A230 ratio exceeding 2.0, confirming minimal contamination. Integrity was confirmed through agarose gel electrophoresis, showing distinct 28S and 18S rRNA bands with a brightness ratio of approximately 2:1, indicative of intact RNA. The 5S rRNA band was visible but less prominent, with all bands appearing sharp and free from smearing. Total RNA content and purity were measured, and cDNA was generated using a commercial reverse transcription kit (G3337, SERVICEBIO, CHINA). Real-time quantitative polymerase chain reaction (PCR) amplification was conducted in a 50 µL reaction system using the SYBR Premixed Ex Taq kit (G3326, SERVICEBIO, CHINA). β-actin served as the endogenous control gene. Relative mRNA levels were assessed utilizing the 2^(-ΔCT) method. The efficiency of all primer pairs was evaluated using the standard curve method, and each primer pair exhibited an efficiency within the optimal range of 90% to 110%. Additionally, melt curve analysis of all amplified products revealed a single peak, indicating high primer specificity and the absence of primer-dimer formation and non-specific amplification. The primer sequences are listed in **Supplementary Table S4**.

### 2.10 Statistical analysis

Data processing and analysis were performed using R software (version 4.3.0; R Project for Statistical Computing, Vienna, Austria). The independent Student’s t-test was used to compare continuous variables in the two groups when the variables followed a normal distribution, unless otherwise specified. For variables that did not follow a Gaussian distribution, the Mann–Whitney U test was conducted. The Kruskal–Wallis test was used to compare three or more groups. Spearman’s correlation analysis was conducted to determine the correlation coefficients between the various molecules. Unless otherwise indicated, statistical significance was evaluated using two-sided p-values, with p-value **<** 0.05 deemed significant.

## 3 Results

### 3.1 Technology roadmap

**Figure 1:**
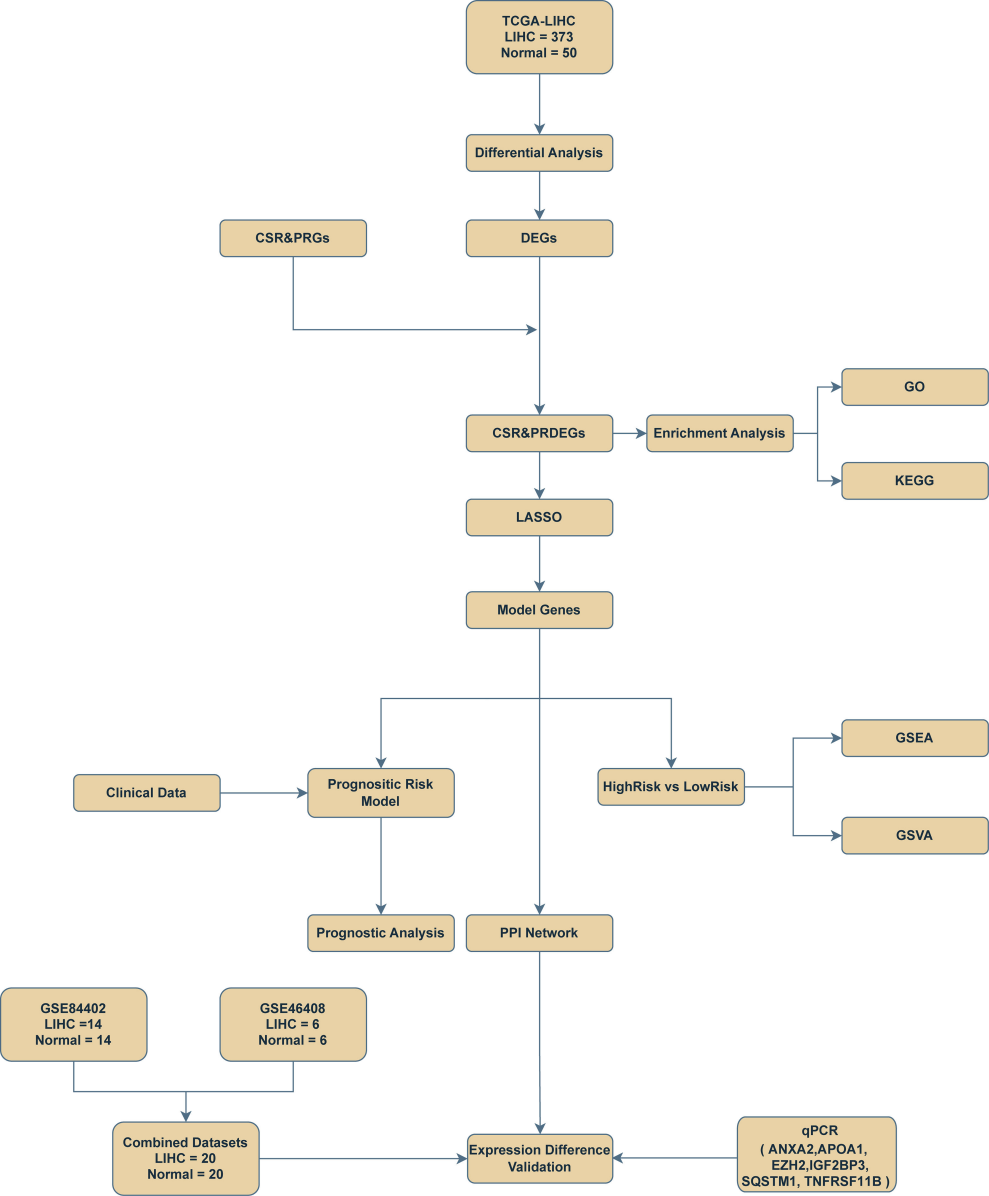
Flow chart for the comprehensive analysis of cellular senescence-related and pyroptosis-related differentially expressed genes. TCGA, The Cancer Genome Atlas; LIHC, liver hepatocellular carcinoma; DEGs, differentially expressed genes; CSR&PRG, cellular senescence-related and pyroptosis-related genes; CSR&PRDEG, cellular senescence-related and pyroptosis-related differentially expressed genes; LASSO, least absolute shrinkage and selection operator; GSEA, gene set enrichment analysis; GSVA, gene set variation analysis; GO, Gene Ontology; KEGG, Kyoto Encyclopedia of Genes and Genomes; qPCR, quantitative polymerase chain reaction.

The technology roadmap is shown in **Figure 1**.

### 3.2 Merging of hepatocellular carcinoma datasets

**Figure 2:**
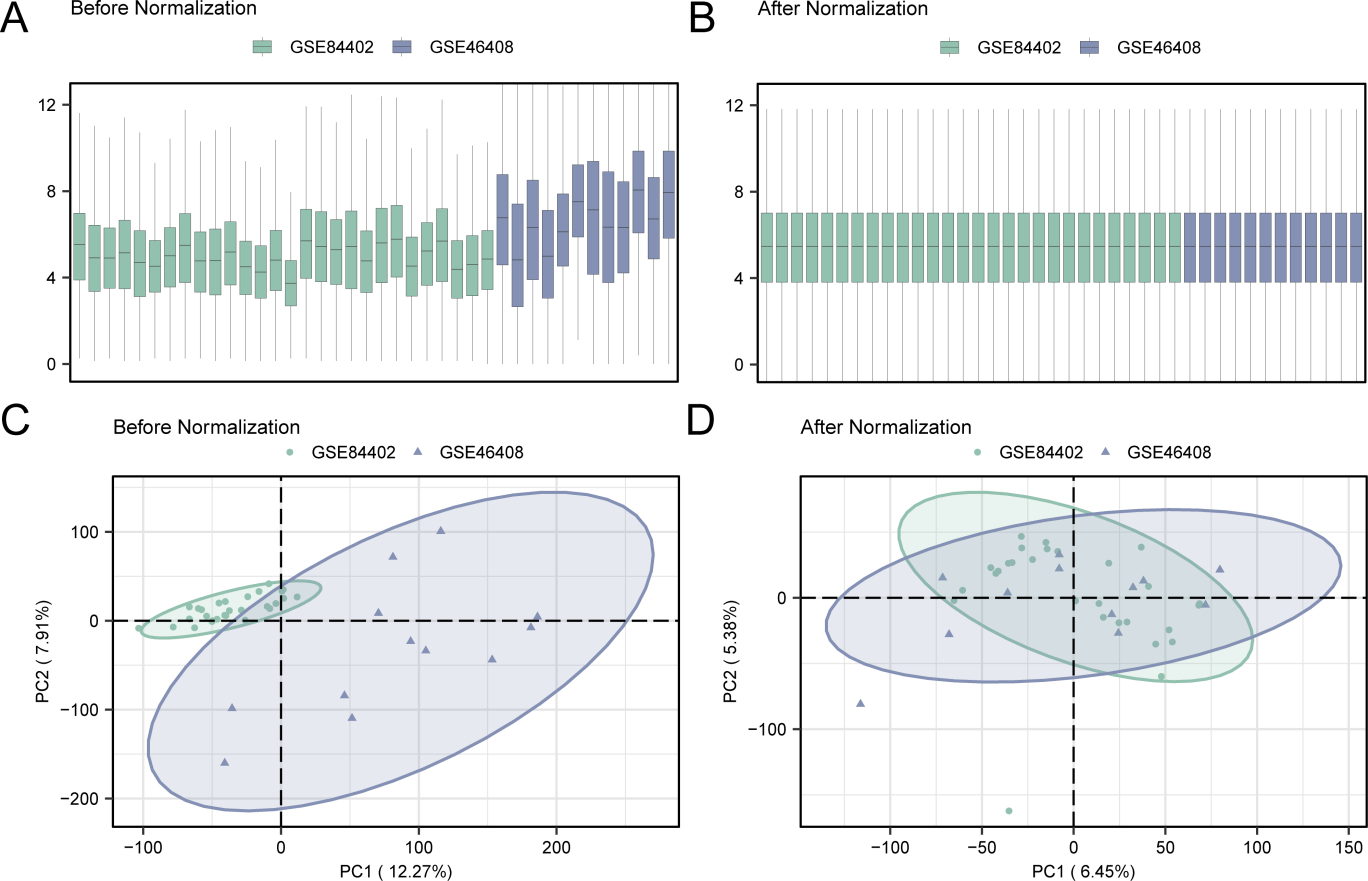
Removal of batch effects for GSE84402 and GSE46408. **(A)** Box plot of combined GEO datasets distribution before batch removal. **(B)** Post-batch integrated GEO datasets (combined dataset) distribution boxplots. **(C)** 2D PCA plot of the datasets before debatching. **(D)** 2D PCA plots of combined GEO dataset after debatching. PCA, principal component analysis. The hepatocellular carcinoma dataset GSE84402 is green, and the hepatocellular carcinoma dataset GSE46408 is blue.

Initially, batch effects were removed from the GSE84402 and GSE46408 datasets using the R package sva, resulting in the combined GEO dataset. The distribution boxplot (**Figures 2A, B**) was first used to compare the expression values of the datasets before and after the removal of batch effects. Subsequently, a PCA plot (**Figures 2C, D**) was used to assess the distribution of the low-dimensional features before and after batch impact removal. The distribution boxplot and PCA plot results indicated that the batch impact in the hepatocellular carcinoma data collection was substantially minimized after its elimination.

### 3.3 Differentially expressed genes related to cell senescence and pyroptosis in hepatocellular carcinoma

**Figure 3:**
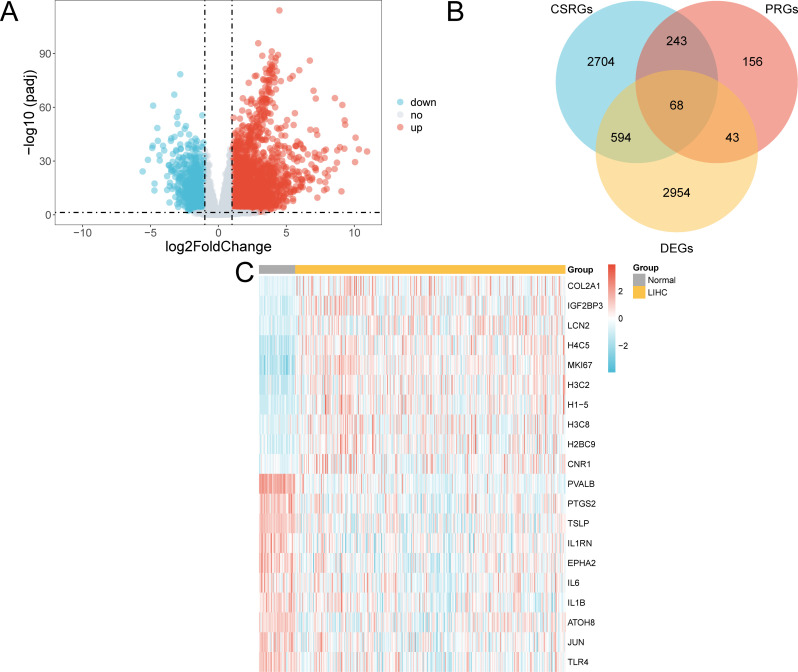
Differential gene expression analysis. **(A)** Volcano plot of gene expression analysis between the LIHC and normal groups in the TCGA-LIHC dataset. **(B)** DEGs and Venn diagrams of CSRGs and PRGs in the TCGA-LIHC dataset. **(C)** Heat map of CSR&PRDEGs in the dataset TCGA-LIHC dataset. TCGA, The Cancer Genome Atlas; LIHC, liver hepatocellular carcinoma; DEGs, differentially expressed genes; CSRGs, cellular senescence-related genes; PRGs, pyroptosis-related genes; CSR&PRDEGs, cellular senescence-related and pyroptosis-related differentially expressed genes. Yellow, LIHC group; grey, normal group. In the heat map, red represents high expression and blue represents low expression, and the depth of the color represents the degree of expression.

The TCGA-LLIHC was divided into LIHC and normal groups. To assess variations in gene expression between these groups within the TCGA-LIHC dataset, differential analysis was conducted using the R package DESeq2.DESeq2 employs a model grounded in the negative binomial distribution, which is well-suited to the count-based structure of sequencing data. It also incorporates size factor normalization to adjust for sequencing depth, thereby ensuring robust and reliable analysis results. This analysis revealed the DEGs between the two groups. The findings showed that in the TCGA-LIHC dataset, 3659 genes complied with |logFC| > 1 and adjusted (adj.) p < 0.05. Within this set of genes, 2,656 were upregulated (logFC > 1 and adj. p < 0.05), whereas 1,003 were downregulated (logFC < -1 and adj. p < 0.05), as shown in the volcano plot (**Figure 3A**).

To identify CSR&PRDEGs, an intersection was performed between DEGs (|logFC| > 1 and adj. p < 0.05), CSRGs, and PRGs. This intersection resulted in 68 DEGs found in both cell senescence and pyroptosis, as detailed in **Supplementary Table S5** and illustrated in **Figure 3B**. The differences in the expression of these CSR&PRDEGs between various sample groups in the TCGA-LIHC dataset were further analyzed, and a heatmap was developed using the R package pheatmap to display the findings (**Figure 3C**).

### 3.4 Creation of a prognostic risk model for hepatocellular carcinoma

**Figure 4:**
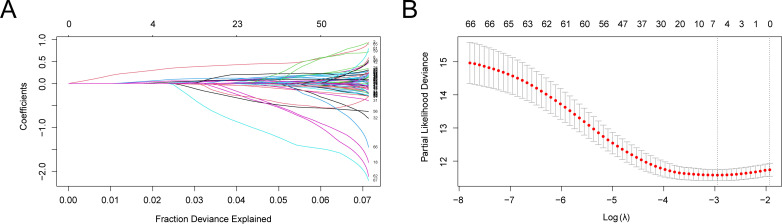
LASSO regression analysis. **(A, B)** Plots of prognostic risk model **(A)** and variable trajectories **(B)** of the LASSO regression model. LASSO, least absolute shrinkage and selection operator. **(A)** illustrates the variation of individual gene coefficients in the LASSO (Least Absolute Shrinkage and Selection Operator) regression analysis. The x-axis represents the Fraction of Deviance Explained, while the y-axis displays the Coefficients for each gene. Distinctly colored lines denote different gene coefficients. As the LASSO process progresses, most gene coefficients gradually diminish to zero, facilitating the selection of significant genes. In this figure, it is evident that as the penalty parameter λ increases (from left to right), the coefficients of certain genes approach zero, indicating their decreasing importance to the model. Ultimately, the retained genes will be utilized to construct prognostic risk models, which are crucial for subsequent analyses. **(B)** presents another critical outcome of the LASSO regression analysis, specifically the relationship between Partial Likelihood Deviance and the penalty parameter λ. The x-axis denotes the Log λ value, while the y-axis indicates the Partial Likelihood Deviance. Red dots represent the deviance for each λ value, and gray vertical lines illustrate the corresponding standard error ranges. It can be observed that as the λ value increases, the partial likelihood deviance decreases, suggesting an improvement in model fit. Additionally, there is a notable inflection point, indicating that at this λ value, the model complexity and predictive power achieve an optimal balance. Selecting an appropriate λ value is essential for establishing the optimal model. Based on information criteria, we can determine the best λ value to finalize gene retention.

To create the LIHC prognostic risk model, 68 CSR&PRDEGs were utilized in a LASSO regression analysis. The analysis was visualized by creating a LASSO regression model map (**Figure 4A**) and a LASSO variable trajectory map (**Figure 4B**). The results indicated that the LASSO regression model included six prognostic risk model genes: *ANXA2, APOA1, EZH2, IGF2BP3, SQSTM1*, and *TNFRSF11B*. The LASSO risk score was determined by employing the specified formula.


$$\begin{array}{c} RiskScore=ANXA2\;*\;\left(0.0548\right)+AP0A1\;*\;\left(-0.0014\right)\\ +EZH2\;*\;\left(0.3410\right)+IGF2BP3\;*\;\left(0.0023\right)\\ +SQSTM1\;*\;\left(0.0879\right)+TNFRSF11B\;*\;\left(0.0299\right) \end{array}$$

Subsequently, LIHC samples from the TCGA-LIHC dataset were classified into high-risk and low-risk groups according to the median LASSO risk score.

### 3.5 Prognostic analysis and validation of a hepatocellular carcinoma prognostic risk model

**Figure 5:**
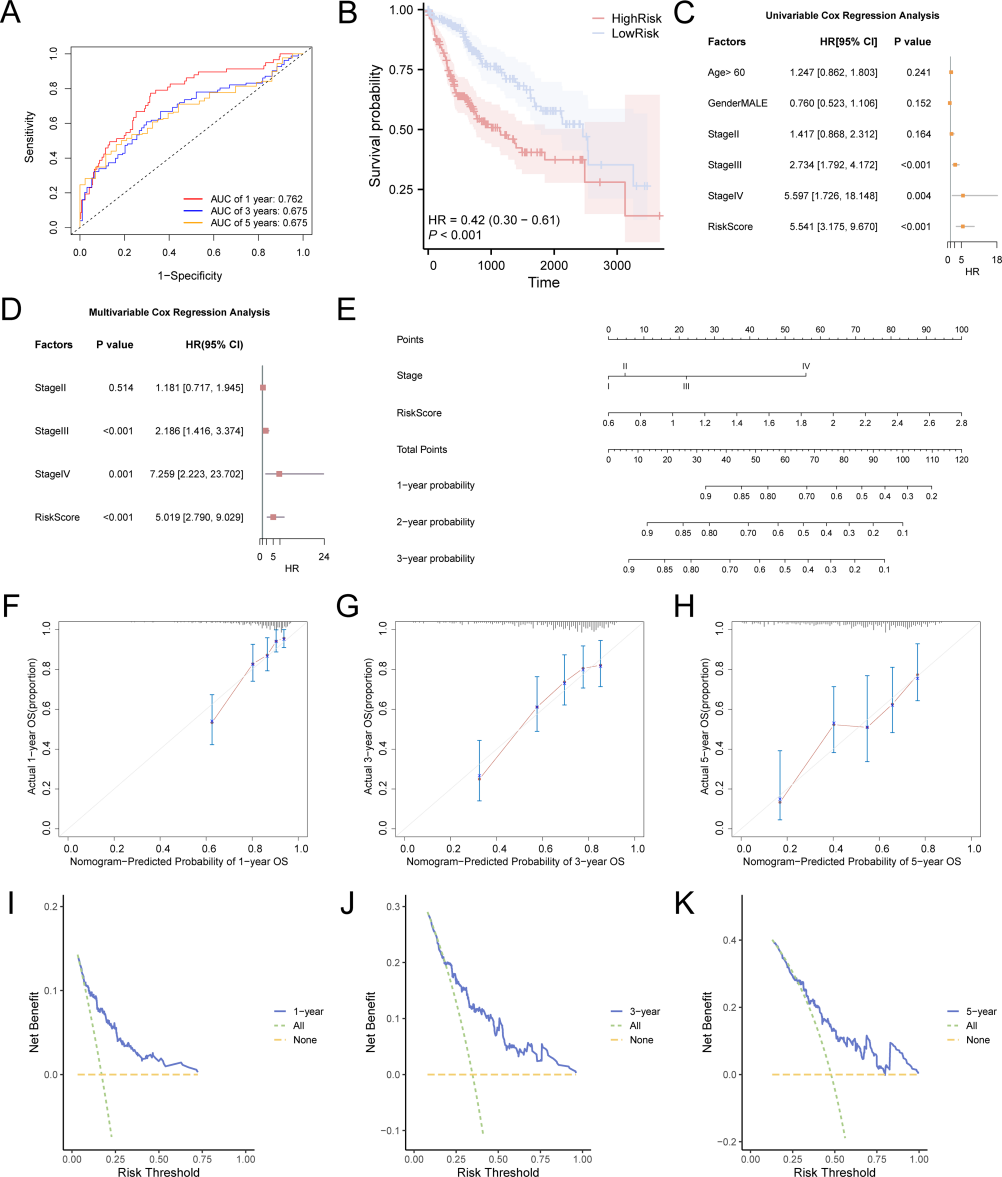
Prognostic model of TCGA-LIHC. **(A)** Time-dependent ROC curves at 1-, 3-, and 5-years for LASSO risk score. **(B)** Prognostic Kaplan–Meier curves between high and low LASSO risk score groups and OS of LIHC. There was a statistically significant difference in survival probability between the high-risk group (red line) and the low-risk group (blue line), indicating that the risk score plays a crucial role in predicting patient prognosis. At 1 year, the survival probability of the high-risk group was markedly lower than that of the low-risk group, demonstrating a substantial survival disparity. At 3 years, although survival remained lower in the high-risk group compared to the low-risk group, the survival gap appeared to have narrowed. By 5 years, this trend persisted: the survival probability in the high-risk group continued to be lower than in the low-risk group, suggesting differences in long-term prognosis. The hazard ratio (HR) was 0.42, indicating that the risk of mortality was 2.38 times higher in the high-risk group compared to the low-risk group. The p-value of less than 0.001 further confirmed that risk scores effectively differentiate survival outcomes between the two groups. **(C)** Forest plot of the univariate Cox regression model based on risk score, age, sex, and clinical stage. **(D)** Forest plot of the prognostic risk model of LIHC based on risk score and clinical stage by multivariate Cox regression analysis. **(E)** Nomogram of the prognostic risk model. **(F-H)** Calibration curves for 1-year **(F)**, 3-years **(G)**, and 5-years **(H)** of the prognostic risk model. In these curves, the horizontal axis indicates the survival probability anticipated by the framework and the vertical axis indicates the actual survival probability. A closer alignment of the predicted line with the ideal gray line reflects better prediction accuracy at that time point. I-K. DCA plot for 1-year **(I)**, 3-years **(J)**, and 5-years **(K)** of the prognostic risk model for LIHC. When the model’s line remains above the “All positive” and “All negative” lines within a particular span, a broader range signifies a higher net benefit and superior model performance. TCGA, The Cancer Genome Atlas; LIHC, liver hepatocellular carcinoma; OS, overall survival; KM, Kaplan–Meier; ROC, receiver operating characteristic; DCA, decision curve analysis. The area under the curve (AUC) has accuracy when between 0.7 and 0.9. Light blue represents the low-risk group and pink represents the high-risk group.

We created time-dependent ROC curves (**Figure 5A**) for LIHC samples from the TCGA-LIHC dataset at 1-, 3-, and 5-year intervals. The findings demonstrated that the LASSO risk score exhibited significant accuracy in predicting prognosis, with the highest predictive performance observed in the first year (AUC = 0.762). Additionally, to assess the diagnostic value of the LASSO risk score for OS, Kaplan–Meier survival analysis was performed using the R package survival, and Kaplan–Meier curves were generated according to the LASSO risk score (**Figure 5B**). The analysis demonstrated a statistically significant variation in OS between the high- and low-risk groups of LIHC samples from the TCGA-LIHC dataset (p < 0.05).

Univariate and multivariate Cox regression analyses were performed to assess the correlation between the LASSO risk score, clinical prognosis, and prognostic ability in LIHC samples of the TCGA-LIHC dataset. Initially, a univariate Cox regression analysis was performed using the LASSO risk score, age, sex, and grade variables. Variables yielding a p-value below 0.10 in the univariate analysis were incorporated into the multivariate Cox regression analysis, and the findings of these analyses were depicted using a forest plot. The findings (**Figures 5C, D**) demonstrated that both the risk score and clinical stage were significant in the univariate analysis (p < 0.10) and remained as independent prognostic factors in the subsequent multivariate Cox regression analysis. The comprehensive results from the univariate and multivariate Cox regression analyses are shown in **Table 3**.

**Table 3:** Results of Univariable and Multivariable Cox Analysis for TCGA-LIHC Datasets.

Characteristics	Total(N)	Univariate analysis		Multivariate analysis	
		Hazard ratio (95% CI)	P value	Hazard ratio (95% CI)	P value
Age	349				
<= 60	171	Reference			
> 60	178	1.247 (0.862 - 1.803)	0.241		
Gender	349				
FEMALE	111	Reference			
MALE	238	0.760 (0.523 - 1.106)	0.152	
Stage	349				
Stage I	173	Reference		Reference	
Stage II	86	1.417 (0.868 - 2.312)	0.164	1.181 (0.717 - 1.945)	0.514
Stage III	85	2.734 (1.792 - 4.172)	**< 0.001**	2.186 (1.416 - 3.374)	**< 0.001**
Stage IV	5	5.597 (1.726 - 18.148)	**0.004**	7.259 (2.223 - 23.702)	**0.001**
RiskScore	349	5.541 (3.175 - 9.670)	**< 0.001**	5.019 (2.790 - 9.029)	**< 0.001**

TCGA, The Cancer Genome Atlas; LIHC, Liver Hepatocellular Carcinoma.

To assess the prognostic value of the risk model for hepatocellular carcinoma more thoroughly, a nomogram was developed that included both the LASSO risk score and clinical stage variables (stage) to demonstrate their relationship (**Figure 5E**). The analysis revealed that the LASSO risk score offered substantially greater utility than clinical stage variables in the hepatocellular carcinoma prognostic risk model.

Furthermore, the calibration of the LIHC prognostic risk framework was evaluated at 1 year (**Figure 5F**), 3 years (**Figure 5G**), and 5 years (**Figure 5H**), with calibration curves created for each time point (**Figures 5F–H**). The results indicated that the time-dependent ROC curve AUC for 1 to 5 years ranged from 0.6 to 0.9, demonstrating the model’s accuracy in prognostic prediction, particularly at the 1-year mark. Decision Curve Analysis (DCA) assessed the clinical utility of the LIHC prognostic risk model at 1 year (**Figure 5I**), 3 years (**Figure 5J**), and 5 years (**Figure 5K**). The 3-year model’s decision curve consistently surpassed the “all positive” and “all negative” lines across a specific risk threshold range, indicating the largest area under the curve and higher net benefit compared to the 1-year and 5-year models. This suggests that the 3-year model offers superior clinical utility for LIHC prediction, with the ranking of predictive effectiveness being: 3 years, 1 year, and 5 years.

### 3.6 Gene ontology and pathway enrichment analysis

**Figure 6:**
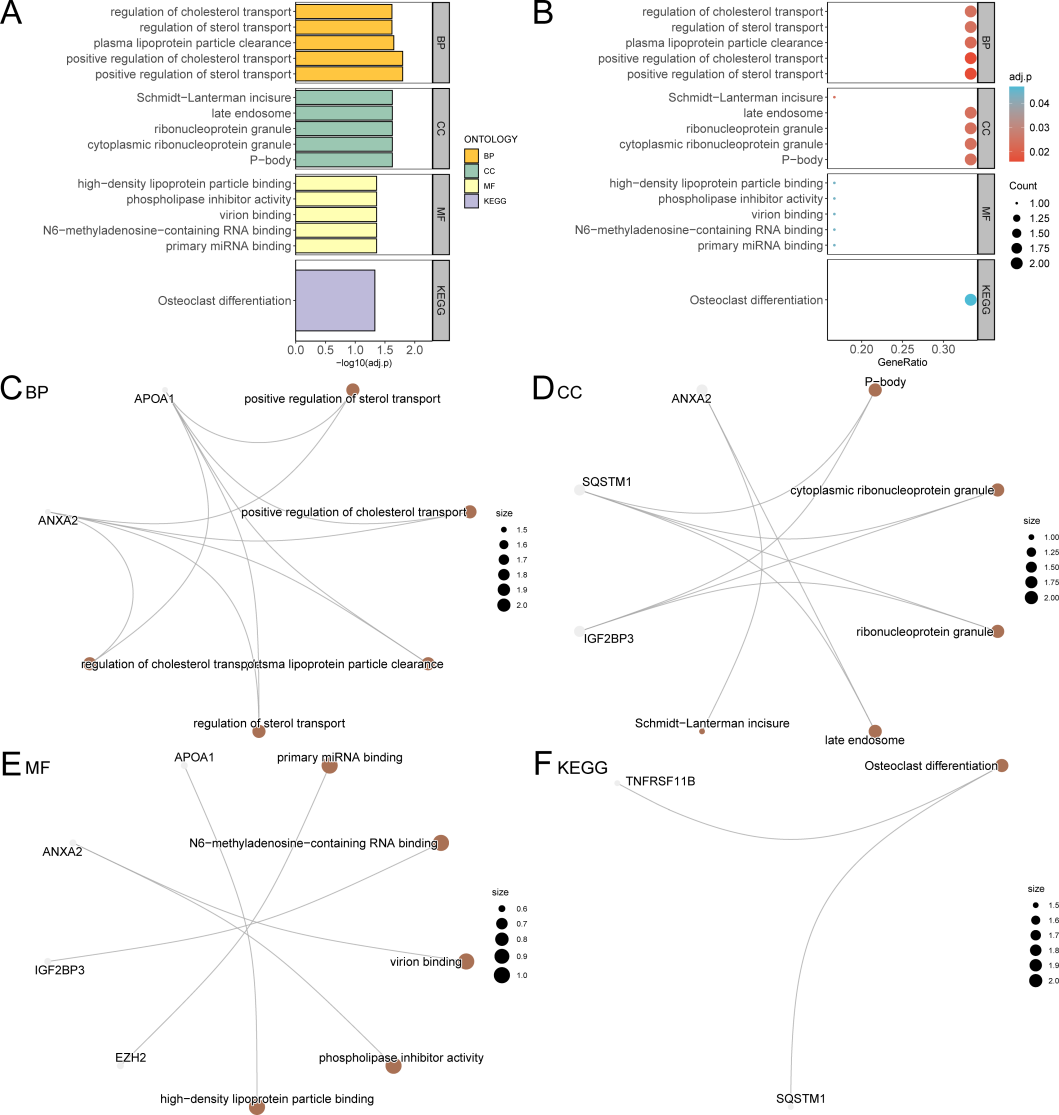
GO and KEGG enrichment analysis for model genes. **(A, B)**. The results of GO and KEGG pathway enrichment analysis of model genes are shown in the bar graph **(A)** and bubble plot **(B)**. GO terms and KEGG terms are shown on the ordinate. **(C-F)** The network diagram of GO and KEGG pathway enrichment analysis results of model genes; BP **(C)**, CC **(D)**, MF **(E)** and KEGG **(F)**. The brown nodes represent items, the gray nodes represent molecules, and the lines represent the relationship between items and molecules. These diagrams depict the relationships between molecules and their respective annotations, with larger nodes indicating entries that include a greater number of molecules. The bubble size in the bubble plot represents the number of genes, and the color of the bubble represents the size of the adj. p-value, the more red the color, the smaller the adj. p-value, and the more blue the color, the larger the adj. p-value. The screening criteria for GO and KEGG pathway enrichment analysis were adj. p < 0.05 and FDR value (q value) < 0.25 were considered statistically significant, with p value correction by the Benjamini-Hochberg method. CSR&PRDEGs, cellular senescence-related and pyroptosis-related differentially expressed genes; GO, Gene Ontology; KEGG, Kyoto Encyclopedia of Genes and Genomes; BP, biological process; CC, cellular component; MF, molecular function; FDR, false discovery rate.

Through GO and KEGG enrichment analyses, we further explored the connection between biological processes, cellular component, molecular function, and biological pathways of the six prognostic risk model genes in LIHC. These six model genes were analyzed for GO and KEGG pathway enrichment, and the detailed findings are presented in **Table 4**. The analysis revealed that these genes were predominantly linked to biological processes, such as positive regulation of sterol transport, positive regulation of cholesterol transport, plasma lipoprotein particle clearance, and other processes related to sterol and cholesterol transport. They were also enriched in cellular components including P-bodies, cytoplasmic ribonucleoprotein granules, ribonucleoprotein granules, late endosomes, and Schmidt-Lanterman incisures. In relation to molecular function, these genes were involved in primary miRNA binding, binding of N6-methyladenosine-containing RNA, virion binding, phospholipase inhibitor activity, and high-density lipoprotein particle binding. Additionally, these genes were significantly present in the biological pathway of osteoclast differentiation. The findings from the GO and KEGG enrichment analyses are depicted using both a bubble plot and bar chart (**Figures 6A, B**).

**Table 4:** Result of GO and KEGG Enrichment Analysis for CSR&PRDEGs.

ONTOLOGY	ID	Description	GeneRatio	BgRatio	pvalue	p.adjust	qvalue
BP	GO:0032373	positive regulation of sterol transport	2/6	38/18614	6.06E-05	1.60E-02	6.25E-03
BP	GO:0032376	positive regulation of cholesterol transport	2/6	38/18614	6.06E-05	1.60E-02	6.25E-03
BP	GO:0034381	plasma lipoprotein particle clearance	2/6	55/18614	1.28E-04	2.24E-02	8.78E-03
BP	GO:0032371	regulation of sterol transport	2/6	79/18614	2.64E-04	2.41E-02	9.43E-03
BP	GO:0032374	regulation of cholesterol transport	2/6	79/18614	2.64E-04	2.41E-02	9.43E-03
CC	GO:0000932	P-body	2/6	98/19518	3.69E-04	2.38E-02	7.65E-03
CC	GO:0036464	cytoplasmic ribonucleoprotein granule	2/6	253/19518	2.43E-03	2.38E-02	7.65E-03
CC	GO:0035770	ribonucleoprotein granule	2/6	271/19518	2.78E-03	2.38E-02	7.65E-03
CC	GO:0005770	late endosome	2/6	296/19518	3.30E-03	2.38E-02	7.65E-03
CC	GO:0043220	Schmidt-Lanterman incisure	1/6	11/19518	3.38E-03	2.38E-02	7.65E-03
MF	GO:0070878	primary miRNA binding	1/6	10/18369	3.26E-03	4.37E-02	1.48E-02
MF	GO:1990247	N6-methyladenosine-containing RNA binding	1/6	10/18369	3.26E-03	4.37E-02	1.48E-02
MF	GO:0046790	virion binding	1/6	11/18369	3.59E-03	4.37E-02	1.48E-02
MF	GO:0004859	phospholipase inhibitor activity	1/6	12/18369	3.91E-03	4.37E-02	1.48E-02
MF	GO:0008035	high-density lipoprotein particle binding	1/6	12/18369	3.91E-03	4.37E-02	1.48E-02
KEGG	hsa04380	Osteoclast differentiation	2/5	135/8659	2.34E-03	4.68E-02	2.96E-02

GO, Gene Ontology; BP, Biological Process; CC, Cellular Component; MF, Molecular Function; KEGG, Kyoto Encyclopedia of Genes and Genomes; CSR&PRDEGs, Cellular Senescence-Related and Pyroptosis-Related Differentially Expressed Genes.

Additionally, network diagrams for biological processes, cellular components, molecular functions, and biological pathways were created based on the GO and KEGG enrichment analyses (**Figures 6C–F**).

### 3.7 Gene set enrichment analysis for high and low risk groups

**Figure 7:**
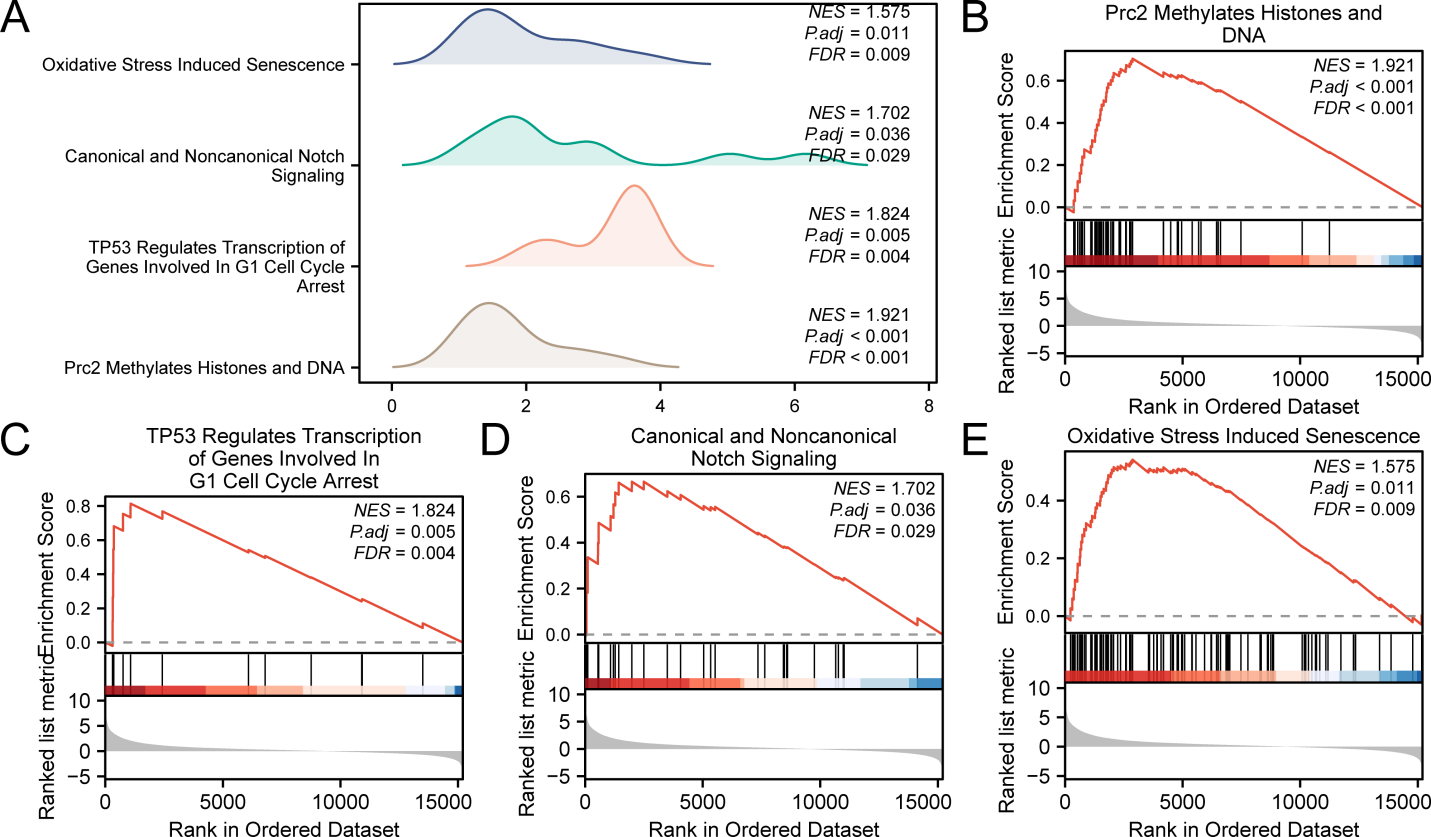
Gene set enrichment analysis for TCGA-LIHC risk groups. **(A)** GSEA four biological function mountain plot display of LIHC samples from the TCGA-LIHC dataset. **(B-E)** GSEA shows that all genes were significantly enriched for Prc2 methylation of histones and DNA **(B)**, TP53 regulates transcription of genes involved in G1 cell cycle arrest **(C)**, canonical and noncanonical notch signaling **(D)**, and oxidative stress-induced senescence **(E)**. The screening criteria for GSEA were adj. p < 0.05 and FDR value (q value) < 0.25, with p value correction by the Benjamini-Hochberg method. TCGA, The Cancer Genome Atlas; LIHC, liver hepatocellular carcinoma; GSEA, gene set enrichment analysis; FDR, false discovery rate.

GSEA was performed to evaluate the impact of gene expression levels on the stratification of LIHC into high-risk and low-risk groups. This analysis examined the relationship between the expression levels of all genes in LIHC samples and their related biological processes, cellular components, and molecular functions. The findings are depicted in a mountain plot (**Figure 7A**) and detailed in **Table 5**. The analysis identified significant enrichment of genes in LIHC samples in processes such as Prc2 methylation of histones and DNA (**Figure 7B**), TP53 regulates the transcription of genes involved in G1 cell cycle arrest (**Figure 7C**), canonical and noncanonical notch signaling (**Figure 7D**), and oxidative stress-induced senescence (**Figure 7E**), among other biological functions and signaling pathways.

**Table 5:** Results of GSEA for TCGA-LIHC Risk Group.

ID	setSize	EnrichmentScore	NES	pvalue	p.adjust	qvalue
PID_PLK1_PATHWAY	40	0.78289	2.09020	2.42E-08	2.94E-06	2.42E-06
REACTOME_ACTIVATED_PKN1_STIMULATES_TRANSCRIPTION_OF_AR_ANDROGEN_RECEPTOR_REGULATED_GENES_KLK2_AND_KLK3	39	0.73692	1.96762	2.42E-06	1.18E-04	9.67E-05
WP_GASTRIC_CANCER_NETWORK_1	25	0.78401	1.96416	6.36E-06	2.60E-04	2.14E-04
REACTOME_RESOLUTION_OF_SISTER_CHROMATID_COHESION	110	0.66768	1.96171	2.45E-10	6.68E-08	5.49E-08
REACTOME_DNA_METHYLATION	39	0.73250	1.95581	3.75E-06	1.77E-04	1.45E-04
REACTOME_MITOTIC_SPINDLE_CHECKPOINT	101	0.67068	1.95341	1.41E-09	2.47E-07	2.03E-07
PID_AURORA_B_PATHWAY	39	0.73126	1.95252	4.01E-06	1.82E-04	1.50E-04
REACTOME_CONDENSATION_OF_PROPHASE_CHROMOSOMES	47	0.71596	1.95189	6.84E-07	4.38E-05	3.60E-05
REACTOME_POLO_LIKE_KINASE_MEDIATED_EVENTS	16	0.84066	1.94058	1.42E-05	4.92E-04	4.04E-04
REACTOME_ASSEMBLY_OF_THE_ORC_COMPLEX_AT_THE_ORIGIN_OF_REPLICATION	37	0.72418	1.92328	2.95E-05	8.61E-04	7.07E-04
REACTOME_PRC2_METHYLATES_HISTONES_AND_DNA	47	0.70481	1.92149	2.09E-06	1.09E-04	8.95E-05
REACTOME_MEIOTIC_RECOMBINATION	60	0.67748	1.89256	2.45E-06	1.18E-04	9.67E-05
REACTOME_SIRT1_NEGATIVELY_REGULATES_RRNA_EXPRESSION	41	0.70152	1.88391	2.68E-05	7.94E-04	6.52E-04
REACTOME_DNA_STRAND_ELONGATION	31	0.72195	1.86609	5.77E-05	1.43E-03	1.17E-03
REACTOME_UNWINDING_OF_DNA	11	0.87994	1.86465	3.78E-05	1.01E-03	8.32E-04
REACTOME_ACTIVATION_OF_THE_PRE_REPLICATIVE_COMPLEX	26	0.73488	1.85837	3.16E-04	5.20E-03	4.27E-03
REACTOME_DEPOSITION_OF_NEW_CENPA_CONTAINING_NUCLEOSOMES_AT_THE_CENTROMERE	46	0.68165	1.85505	2.45E-05	7.50E-04	6.16E-04
REACTOME_TP53_REGULATES_TRANSCRIPTION_OF_GENES_INVOLVED_IN_G1_CELL_CYCLE_ARREST	14	0.81389	1.82366	3.02E-04	5.12E-03	4.21E-03
WP_CANONICAL_AND_NONCANONICAL_NOTCH_SIGNALING	27	0.66562	1.70227	4.10E-03	3.58E-02	2.95E-02
REACTOME_OXIDATIVE_STRESS_INDUCED_SENESCENCE	92	0.54154	1.57475	8.02E-04	1.07E-02	8.79E-03

GSEA, Gene Set Enrichment Analysis; TCGA, The Cancer Genome Atlas; LIHC, Liver Hepatocellular Carcinoma.

### 3.8 Gene set variation analysis for high- and low-risk groups

**Figure 8:**
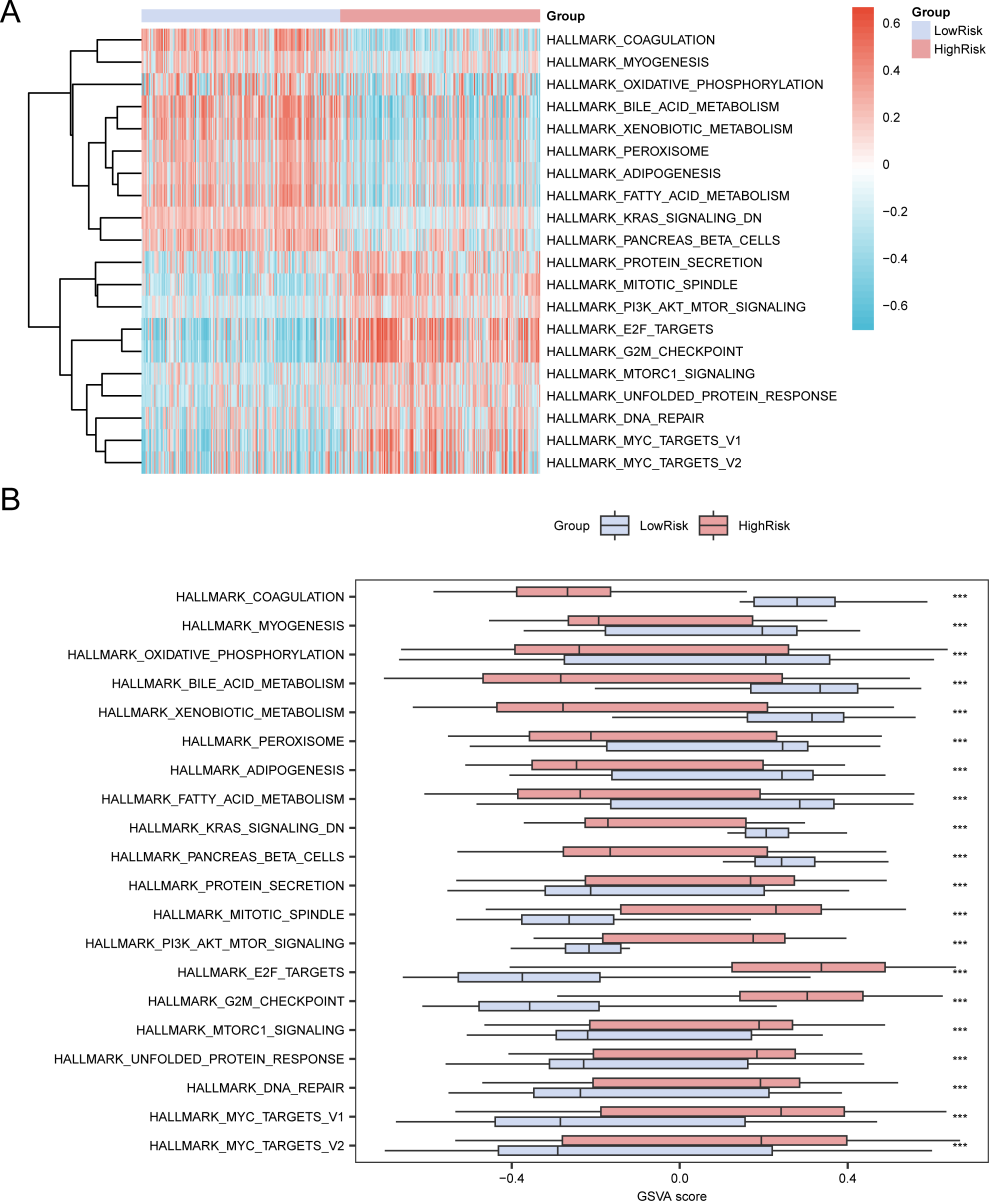
Gene set variation analysis for TCGA-LIHC risk groups. Heat map **(A)** and group comparison map **(B)** of GSVA results of the high-risk and low-risk groups of LIHC samples in the TCGA-LIHC dataset. TCGA, The Cancer Genome Atlas; LIHC, liver hepatocellular carcinoma; GSVA, gene set variation analysis. *** p value < 0.001. High-risk group, pink; low-risk group, light blue. Blue shows low enrichment and red shows high enrichment in the heat map. The screening criteria for GSVA was adj. p < 0.05, with p value correction by the Benjamini-Hochberg method.

GSVA was conducted on all genes in the LIHC samples of the TCGA-LIHC dataset; detailed results are presented in **Table 6**. Positive enrichment pathways with adj. p < 0.05, the top 10 logFC rankings, and the top 10 negative enrichment pathways were identified. A heat map (**Figure 8A**) was used to visualize the altered gene expression of these 20 pathways in the high- and low-risk groups.

**Table 6:** Results of GSVA for TCGA-LIHC Risk Groups.

ID	logFC	AveExpr	t	P.Value	adj.P.Val	B
HALLMARK_COAGULATION	0.396871	0.002881	14.07214	1.23E-36	1.53E-35	72.15239
HALLMARK_BILE_ACID_METABOLISM	0.382569	0.027628	11.27163	1.09E-25	7.79E-25	47.07474
HALLMARK_XENOBIOTIC_METABOLISM	0.364831	0.018406	11.58021	7.64E-27	6.37E-26	49.71626
HALLMARK_FATTY_ACID_METABOLISM	0.310226	0.014717	10.10934	1.74E-21	9.69E-21	37.46812
HALLMARK_ADIPOGENESIS	0.268789	0.004818	9.798618	2.10E-20	8.75E-20	35.00108
HALLMARK_KRAS_SIGNALING_DN	0.257042	0.051305	14.45349	3.39E-38	5.66E-37	75.725
HALLMARK_PANCREAS_BETA_CELLS	0.254193	0.069833	10.2891	4.05E-22	2.53E-21	38.91581
HALLMARK_PEROXISOME	0.224815	0.016032	7.733553	9.07E-14	3.02E-13	19.89749
HALLMARK_OXIDATIVE_PHOSPHORYLATION	0.188229	-0.02831	5.237335	2.68E-07	6.08E-07	5.31765
HALLMARK_MYOGENESIS	0.178348	0.008016	7.341872	1.25E-12	3.89E-12	17.31888
HALLMARK_PROTEIN_SECRETION	-0.15885	-0.02176	-5.50414	6.75E-08	1.61E-07	6.653213
HALLMARK_DNA_REPAIR	-0.18869	-0.0277	-6.60549	1.31E-10	3.44E-10	12.7505
HALLMARK_UNFOLDED_PROTEIN_RESPONSE	-0.20189	-0.04308	-7.77574	6.80E-14	2.43E-13	20.18114
HALLMARK_MTORC1_SIGNALING	-0.20734	-0.02473	-8.06065	9.46E-15	3.64E-14	22.12588
HALLMARK_PI3K_AKT_MTOR_SIGNALING	-0.21079	-0.03681	-9.84404	1.46E-20	6.65E-20	35.35885
HALLMARK_MYC_TARGETS_V2	-0.22072	-0.03908	-5.98397	4.95E-09	1.24E-08	9.196559
HALLMARK_MYC_TARGETS_V1	-0.32392	-0.02969	-9.92122	7.91E-21	3.95E-20	35.96902
HALLMARK_MITOTIC_SPINDLE	-0.34664	-0.02638	-13.3943	6.67E-34	6.67E-33	65.88139
HALLMARK_G2M_CHECKPOINT	-0.5273	-0.02842	-19.3794	4.99E-59	2.50E-57	123.547
HALLMARK_E2F_TARGETS	-0.54227	-0.02069	-17.4787	6.85E-51	1.71E-49	104.8589

GSVA, Gene Set Variation Analysis; TCGA, The Cancer Genome Atlas; LIHC, Liver Hepatocellular Carcinoma.

The differences were further confirmed using the Mann–Whitney U test, and the results are illustrated in a comparative diagram for the groups (**Figure 8B**). GSVA revealed that several pathways, including Myc targets v2, Myc targets v1, DNA repair, E2F targets, PI3K-AKT-mTOR signaling, mitotic spindle, protein secretion, bile acid metabolism, oxidative phosphorylation, myogenesis, adipogenesis, and coagulation, demonstrated statistically significant disparities between the high- and low-risk groups (p < 0.05).

### 3.9 Protein interaction network construction and differential gene expression verification of the prognostic risk model

**Figure 9:**
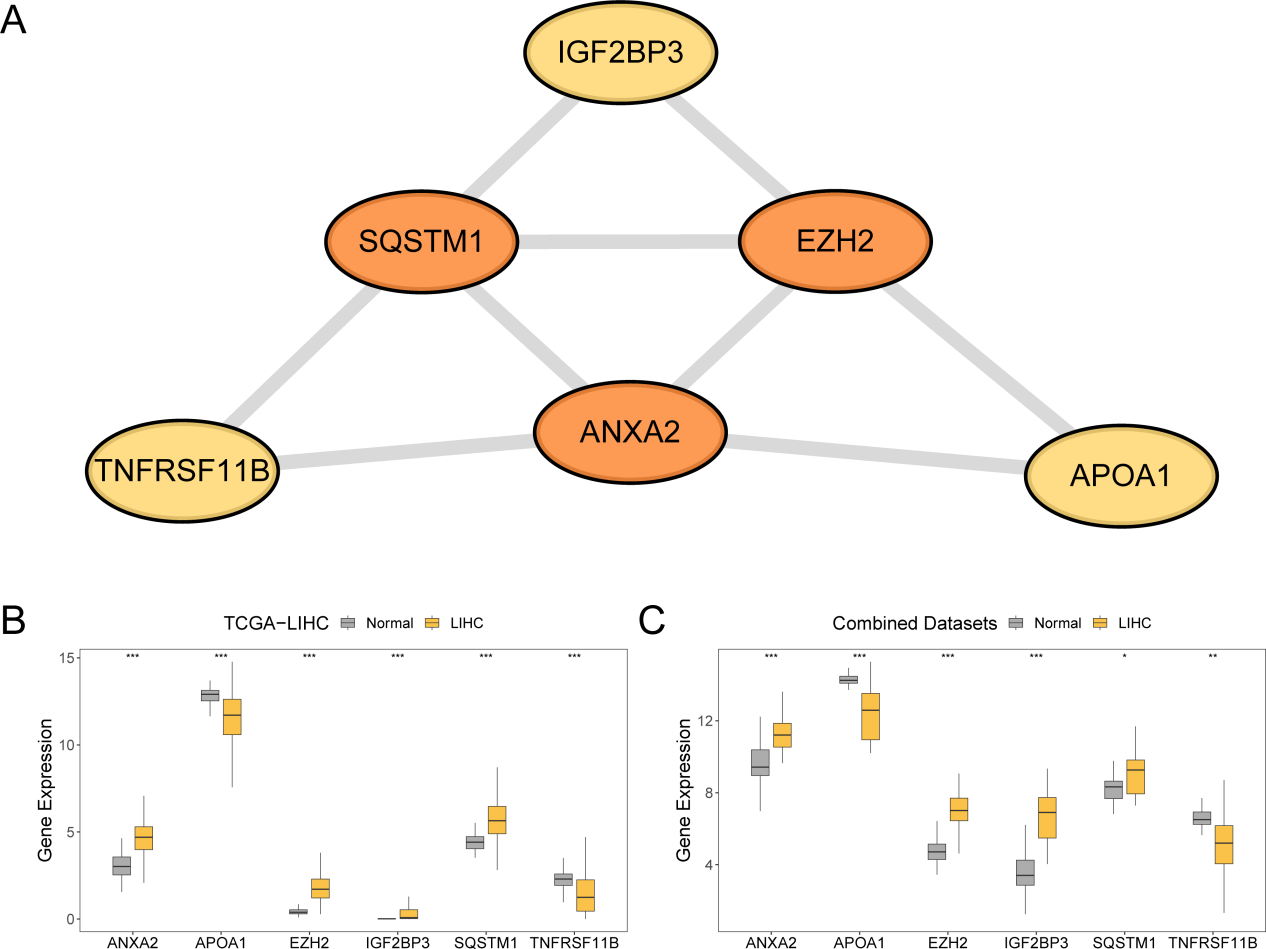
Protein-protein interaction network and differential expression validation. **(A)** PPI network of prognostic risk model genes calculated from the STRING database. **(B, C)** Group comparison of expression difference of model genes in LIHC and normal groups in the TCGA-LIHC and combined datasets. TCGA, The Cancer Genome Atlas; LIHC, liver hepatocellular carcinoma; PPI, protein-protein interaction. ***p value < 0.001; **p value < 0.01; *p value < 0.05. In the group comparison plot, yellow represents the LIHC group and grey represents the normal group.

Initially, a PPI analysis was conducted, and the PPI network for the six prognostic risk model genes was constructed using the STRING database (**Figure 9A**). These findings demonstrate that the six model genes related to prognostic risk were *ANXA2, APOA1, EZH2, IGF2BP3, SQSTM1*, and *TNFRSF11B*.

To explore the differences in the expression of prognostic risk model genes between the LIHC and normal groups within the TCGA-LIHC dataset and the combined dataset, group comparison plots were employed. The differential results (**Figures 9B, C**) revealed that the expression levels of the six genes in the prognostic risk framework between the LIHC and normal groups were statistically significant (p < 0.001). These genes included *ANXA2, APOA1, EZH2, IGF2BP3, SQSTM1*, and *TNFRSF11B*. Additionally, the expression levels of four prognostic risk model genes (*ANXA2, APOA1, EZH2*, and *IGF2BP3*) in the combined dataset of the LIHC and normal groups were significantly different (p < 0.001). The expression levels of *TNFRSF11B* in the combined dataset demonstrated a significant difference between the LIHC and normal groups (p < 0.01), whereas the expression of *SQSTM1* in combined dataset was statistically significant (p < 0.05).

### 3.10 mRNA expression of ANXA2, APOA1, EZH2, IGF2BP3, SQSTM1, and TNFRSF11B

**Figure 10:**
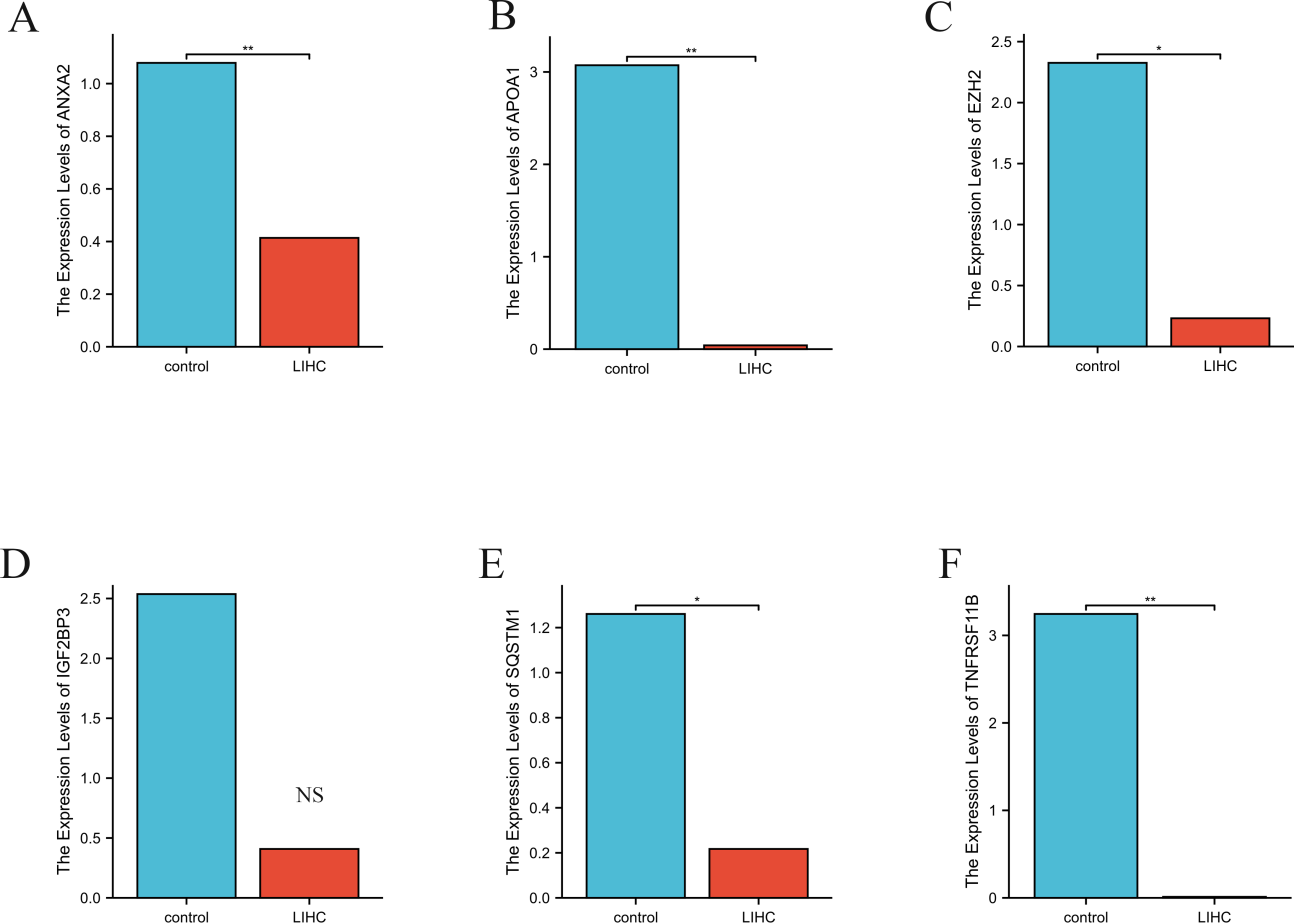
The relative expression levels of *ANXA2* **(A)**, *APOA1* **(B)**, *EZH2* **(C)**, *IGF2BP3* **(D)**, *SQSTM1* **(E)** and *TNFRSF11B* **(F)**, in control and LIHC samples identified by RT-PCR. Actin was used as a reference. RT-PCR, reverse transcription polymerase chain reaction. **p value < 0.01; *p value < 0.05; NS non-significant.

The *ANXA2* expression levels (**Figure 10A**) were significantly lower in the LIHC group than in the control group (p < 0.01). In contrast, *APOA1* (**Figure 10B**) showed markedly higher expression in the LIHC group than in the control group (p < 0.01). Similarly, *EZH2* (**Figure 10C**) showed substantially elevated expression in the LIHC group relative to that in the control group (p < 0.05). Although *IGF2BP3* (**Figure 10D**) exhibited higher expression levels in the LIHC group compared to the control group, the difference was not statistically significant. The expression of *SQSTM1* (**Figure 10E**) was substantially higher in the LIHC group than the control group (p < 0.05). Finally, the expression of *TNFRSF11B* (**Figure 10F**) was significantly elevated in the LIHC group compared to the control group (p < 0.01).

## 4 Discussion

LIHC is a common cancer that has a significant impact on morbidity and mortality (37). The high mortality rate associated with LIHC primarily stems from its late diagnosis and the scarcity of effective treatment options, highlighting the urgent need for novel diagnostic and therapeutic strategies (38). The progression of LIHC is influenced by numerous genetic and environmental factors (39, 40). Chronic inflammation underpins cancer initiation and progression (41–43). Chronic inflammation is closely linked to cell senescence and pyroptosis (44, 45) and can precipitate tumor formation. Thus, elucidating the molecular mechanisms of LIHC through cell senescence and pyroptosis is crucial for its early detection, effective treatment, and enhanced patient outcomes. However, the research in this field is limited.

Given the pressing need to enhance diagnostic and therapeutic strategies for LIHC, our research focused on genes linked to inflammatory cell senescence and pyroptosis, identifying potential markers for early diagnosis and treatment and developing prognostic models. Biomarkers hold significant potential in clinical applications. First, the six model genes identified through LASSO regression (ANXA2, APOA1, EZH2, IGF2BP3, SQSTM1, TNFRSF11B) demonstrate robust prognostic capabilities. Evaluating the correlation between the LASSO Risk Score and patient survival can help clinicians identify high-risk patients and personalize treatment plans. Second, these genes are closely associated with the onset and progression of hepatocellular carcinoma, making them promising candidates for early diagnosis and disease monitoring. Assessing their expression levels can aid in determining disease severity and guiding therapeutic decisions. Third, investigating the molecular mechanisms of these genes can identify novel therapeutic targets. For example, understanding EZH2’s role in cell proliferation and tumor progression may lead to specific inhibitors targeting this gene. Finally, leveraging risk scores from these model genes facilitates personalized clinical management. Patients in different risk categories may require distinct follow-up frequencies and treatment approaches, optimizing care and enhancing outcomes.

We found six key genes: *ANXA2, APOA1, EZH2, IGF2BP3, SQSTM1*, and *TNFRSF11B*. Kaplan–Meier survival analysis and ROC curves were used to evaluate the model’s predictive performance. The findings revealed a substantial variation in OS between the high- and low-risk groups (p < 0.05). The model demonstrated high accuracy in predicting survival at 1-year, 3-year, and 5-year intervals in patients with LIHC. The expression levels of these genes were significantly elevated in the peripheral blood of patients with LIHC compared to normal controls (p < 0.05). Functional enrichment analysis identified important biological pathways and processes associated with these genes, providing insights into the molecular mechanisms that drive LIHC progression. This study utilized multiple datasets to explore the gene-disease relationship from various perspectives, constructing a prognostic risk model based on CSR&PRDEGs to enhance prognostic accuracy for LIHC, and examining the biological functions and potential mechanisms of these genes in LIHC.

ANXA2 is a calcium-dependent membrane-binding protein involved in apoptosis, proliferation, and migration (46–48). In hepatocellular carcinoma (HCC), ANXA2 overexpression correlates with tumor aggressiveness and metastasis, influencing cancer progression by regulating the extracellular matrix and promoting tumor cell migration and invasion (49, 50). Additionally, ANXA2 interacts with inflammatory response and survival signaling pathways, contributing to the development of liver cancer (51, 52). Regarding pyroptosis, ANXA2 modulates the tumor microenvironment through the β-catenin signaling pathway, linked to pro-inflammatory factors. In cellular senescence, ANXA2 mediates responses to DNA damage and may affect the transformation of tumor cells into a drug-resistant state by regulating the regenerative potential of senescent cells. High ANXA2 expression is associated with poor prognosis in several cancers, underscoring its role in tumor escape mechanisms.

EZH2, an important epigenetic regulator, silences gene expression and is associated with HCC proliferation, migration, and drug resistance (53). By downregulating tumor suppressor genes, EZH2 enhances HCC malignancy and may serve as a potential therapeutic target. Additionally, EZH2 inhibits hepatocyte aging and promotes tumor cell survival, potentially facilitating tumor progression by modulating inflammatory responses. The interaction between ANXA2 and EZH2 in cellular senescence and pyroptosis merits further investigation, as ANXA2’s pro-inflammatory effects may influence tumor therapy responses.

SQSTM1, involved in autophagy and antioxidant response, is often overexpressed in HCC, contributing to tumor cell survival and proliferation (54). It plays a role in hepatocellular inflammation, oxidative stress, and metabolism by inhibiting apoptosis. TNFRSF11B is crucial in immune responses and tumorigenesis, influencing the tumor microenvironment through the RANK-RANKL signaling pathway, which promotes tumor cell proliferation and metastasis. Its expression correlates positively with HCC aggressiveness, likely due to interactions with local immune cells that suppress anti-tumor immunity. Although recognition of TNFRSF11B’s clinical significance is growing, research on it as a therapeutic target remains limited. Preliminary studies suggest that blocking TNFRSF11B signaling can enhance chemotherapy sensitivity and reduce recurrence rates in HCC. Therefore, exploring TNFRSF11B in HCC therapy, especially in combination with immunotherapies and targeted treatments, is critical. Studies on its role in other cancers, such as breast and lung cancer, provide insights supporting its evaluation in HCC, with inhibition in breast cancer models significantly delaying tumor growth and metastasis. Future research should focus on elucidating TNFRSF11B’s functional mechanisms in HCC and its therapeutic potential as a target.

Potential Value of Biomarkers in Clinical Applications: Prognostic Ability: Six model genes (ANXA2, APOA1, EZH2, IGF2BP3, SQSTM1, TNFRSF11B) identified through LASSO regression analysis exhibit robust prognostic capabilities. Evaluating the correlation between the LASSO Risk Score and patient survival outcomes can assist clinicians in identifying high-risk patients and subsequently personalizing treatment plans. Biomarker Development: The expression levels of these model genes are significantly associated with the onset and progression of hepatocellular carcinoma, making them potential biomarkers for early diagnosis and disease monitoring. Assessing these genes can aid in determining disease severity and guiding therapeutic decisions. Basis for Targeted Therapy: In-depth investigation into the molecular mechanisms of these prognostic model genes can facilitate the identification of novel targets and advance the development of targeted therapies for hepatocellular carcinoma. For instance, elucidating the role of EZH2 in cell proliferation and tumor progression may lead to the creation of inhibitors targeting this protein. Personalized Medicine: Leveraging the risk scores derived from these model genes enables personalized clinical management. Patients in varying risk categories may require different follow-up frequencies and treatment strategies, thereby optimizing patient care and enhancing treatment outcomes.

The enrichment analysis results of this study offer significant insights into the biological processes and pathways associated with prognostic risk model genes for LIHC. Through GO and KEGG enrichment analyses, we recognized that six prognostic risk model genes (*ANXA2, APOA1, EZH2, IGF2BP3, SQSTM1*, and *TNFRSF11B*) were strongly linked to various biological processes, cellular components, and molecular functions. Specifically, these genes are involved in critical processes such as positive regulation of sterol transport and P-body formation, both of which are vital for maintaining cellular homeostasis and responding to stress (55). Additionally, GSEA detected significant enrichment in various pathways such as PRC2-mediated histone and DNA methylation and TP53 regulation of G1 cell cycle arrest genes (56). The PRC2 complex plays a role in epigenetic regulation via histone methylation, which, in turn, influences gene expression and cell differentiation. The TP53 pathway is a well-established tumor suppressor that governs cell cycle arrest, apoptosis, and DNA repair, thereby preventing cancer progression (57). Furthermore, GSVA highlighted notable variations in pathways including MYC v2 and DNA repair between high- and low-risk groups. The MYC pathway is a key regulator of cellular growth and proliferation, and is frequently dysregulated in cancer, contributing to uncontrolled cell division and tumor development. DNA repair mechanisms are essential for preserving genomic integrity, and their disruption can lead to increased mutation rates and cancer (58). PPI network analysis demonstrated that these prognostic genes are interconnected and involved in immune regulation. For example, *EZH2* suppresses T cell infiltration and function, thereby fostering an immunosuppressive tumor microenvironment (59). Similarly, *TNFRSF11B* modulates immune responses by interacting with the RANK/RANKL pathway, which is vital for dendritic and T cell function (60). The enrichment of these genes in immunomodulatory pathways, including the TP53 pathway and MYC targets, underscores their role in influencing immune responses in LIHC (61, 62). These interactions suggest a collaborative contribution of these genes to the prognostic model, highlighting their potential as therapeutic targets (63).

Cellular senescence is an irreversible state marked by loss of proliferative capacity and significant phenotypic changes, including the secretion of senescence-associated secretory phenotype (SASP) molecules, which impact the immune microenvironment in hepatocellular carcinoma (HCC) (44, 64). SASP components can recruit immune cells such as macrophages, T cells, and natural killer (NK) cells, promoting local inflammation and activating the immune response (65). However, the persistence of senescent cells may foster immune tolerance, allowing tumor cells to evade detection. Key aging-related genes like p16INK4a, p21CIP1/WAF1, and IL-6 are upregulated in HCC, stimulating cytokine release and influencing immune cell infiltration.

Pyroptosis, a form of inflammatory programmed cell death, also plays a critical role in HCC. It induces immune responses by releasing inflammatory factors like IL-1β and IL-18. While activation of pyroptosis-related genes (e.g., CASP1, GSDMD) enhances inflammation and attracts immune cells, it may paradoxically promote tumor progression by allowing HCC cells to escape immune surveillance through modulation of apoptosis-inducing factors (66, 67).

There exists a complex interplay between cellular senescence and pyroptosis, whereby senescent cells can stimulate pyroptosis, further exacerbating immunosuppression and diminishing the functionality of tumor-infiltrating immune cells, facilitating immune evasion. The MAPK/ERK pathway is crucial in this context, as it drives both senescence and pyroptosis, influencing HCC progression.

In hepatitis C virus (HCV) infections, cell senescence exacerbates liver damage and contributes to HCC development through inflammation, pyroptosis, and senescence mechanisms. Viral genotype and host genetic factors, particularly single nucleotide polymorphisms (SNPs), modulate the expression of genes like ANXA2 and SQSTM1, impacting immune escape and inflammation intensity.

In high-prevalence regions, targeted antiviral therapy for high-risk populations is essential. Monitoring key gene expressions can identify individuals at increased risk for HCC, laying the groundwork for precision medicine. Reducing viral load through antiviral treatment not only inhibits viral replication but also mitigates pyroptosis and senescence associated with chronic inflammation, lowering HCC incidence.

In summary, exploring the connections between HCV-induced inflammation, pyroptosis, cellular senescence, and the influence of viral genotypes and host genetic factors is vital for understanding HCC pathogenesis. This research will inform targeted antiviral therapies and risk monitoring strategies for high-risk populations.

Although this study presented promising results, several limitations must be considered. First, these findings are primarily derived from bioinformatic analyses and lack validation through comprehensive wet laboratory experiments. Additionally, the study does not integrate pathway-level correlations with clinical variables such as tumor stage or grade, which would provide deeper insights into the underlying biological mechanisms. Second, although the sample size was considerable, it may not fully represent the diversity within LIHC. Third, the lack of clinical validation restricts the immediate practical application of the prognostic risk model in clinical settings. Finally, combining the datasets from various sources introduces batch effects. In the future, we plan to undertake a series of comprehensive studies: 1. Mechanism Research: We will conduct an in-depth investigation into the biological functions and mechanisms of these model genes in hepatocellular carcinoma (HCC), focusing on the regulation of cell signaling pathways and the construction of interaction networks. This will help us determine their specific roles in cancer initiation and progression. 2. Large-Scale Clinical Validation: We aim to expand our sample size to include patients from diverse populations and disease stages, ensuring the applicability and generalizability of our model. Additionally, we will evaluate its performance across various clinical settings. 3. Integration with Other Clinical Factors: We will explore the combination of model genes with traditional clinical predictors (e.g., age, gender, clinical stage) to develop a more comprehensive prognostic model, thereby enhancing predictive accuracy. 4. Functional Studies: Using cell lines and animal models, we will investigate the functional roles of these genes in HCC, validate their significance in hepatocarcinogenesis, and assess their potential as therapeutic targets. 5. Drug Sensitivity Analysis: We will evaluate the sensitivity of different drugs related to the model genes, providing a foundation for targeted therapy in HCC. Furthermore, we will explore whether specific drugs can yield better treatment outcomes for high-risk patient groups.

## 5 Conclusion

The development and progression of hepatocellular carcinoma (HCC) are influenced by a multitude of mechanisms, including alterations in the tumor microenvironment, immune evasion, and modulation of cell signaling pathways. Research has demonstrated that the tumor microenvironment not only impacts tumor cell proliferation but also facilitates liver cancer progression by regulating local immune responses. The chronic inflammatory state within the liver can lead to immune cell dysfunction, thereby creating a conducive environment for cancer cell growth. Moreover, HCC cells can evade host immune surveillance through various mechanisms, leading to the suppression of T cell activity. These mechanisms interact in a complex and mutually reinforcing manner, collectively driving the development and progression of HCC. Comparative Analysis In our study, an analysis of 68 differentially expressed genes associated with cell senescence and pyroptosis (CSR&PRDEGs) revealed trends consistent with previous research. Notably, the expression levels of certain genes, such as ANXA2 and EZH2, were significantly elevated in HCC tumor tissues, further underscoring their importance in hepatocellular carcinoma development. Unlike other studies, we observed that the expression patterns of these immune-related genes may influence the characteristics of the tumor microenvironment, offering new insights into the pathogenesis of HCC. Relationship of Treatment Strategies Our findings suggest a potential new therapeutic target for hepatocellular carcinoma (HCC). Specifically, ANXA2 and EZH2 have been identified as key regulators in the proliferation and metastasis of liver cancer. Targeting these genes can enhance existing targeted therapies and immunotherapies. For instance, inhibiting EZH2 activity may restore immune cell function and improve the efficacy of immunotherapy for liver cancer. Moreover, the identification of novel biomarkers such as TNFRSF11B provides a foundation for assessing patient prognosis and developing personalized treatment strategies. Future Research Directions Based on our findings, future studies should delve into the specific roles of cell senescence and pyroptosis in HCC mechanisms. It is crucial to evaluate the clinical validation potential of proposed biomarkers in both immunotherapy and targeted therapy contexts. Additionally, clinical trials should be conducted in combination with other treatments to explore optimal treatment strategies.

## Data Availability

The datasets presented in this study can be found in online repositories. The names of the repository/repositories and accession number(s) can be found in the article/**Supplementary Material**.
