# Supplementary material for: Creation of an innovative diagnostic framework for hepatocellular carcinoma employing bioinformatics techniques focused on senescence-related and pyroptosis-related genes
Source: Front Oncol. 2025 Feb 13;15:1485421. doi: 10.3389/fonc.2025.1485421 (PMC11864952; doi:10.3389/fonc.2025.1485421)
Supplement: Supplementary file 4 [file DataSheet4.docx]

**Table S4 Primers used for RT-PCR in this study**

| **Gene** | **Forward primer(5′-3′)** | **Reverse primer(5′-3′)** |
| --- | --- | --- |
| ANXA2 | ATATTGCCTTCGCCTACCAGAG | GCGTCATACTGAGCAGGTGTCTT |
| IGF2BP3 | ATCTGAACGCCTTGGGTCTG | ATTGCTCAAACTGCGGGTAG |
| SQSTM1 | CCCTCTGGGCATTGAAGTTGAT | CTCTTCTCCTCTGTGCTGGAAC |
| APOA1 | GGGATAACCTGGAAAAGGAGACA | CTCCATCTCCTCCTGCCACTT |
| TNFRSF11B | CCTTGCCCTGACCACTACTACA | CCTTGCCCTGACCACTACTACA |
| EZH2 | TCCAACACAAGTCATCCCATTA | GGGCATTCACCAACTCCAC |
| ACTIN | CACCCAGCACAATGAAGATCAAGAT | CCAGTTTTTAAATCCTGAGTCAAGC |
